# Supplementary material for: MALDI-TOF MS as a method for rapid identification of Phytophthora de Bary, 1876
Source: PeerJ. 2021 Jul 19;9:e11662. doi: 10.7717/peerj.11662 (PMC8297470; doi:10.7717/peerj.11662)
Supplement: Supplemental Information 1 [file peerj-09-11662-s001.pdf]

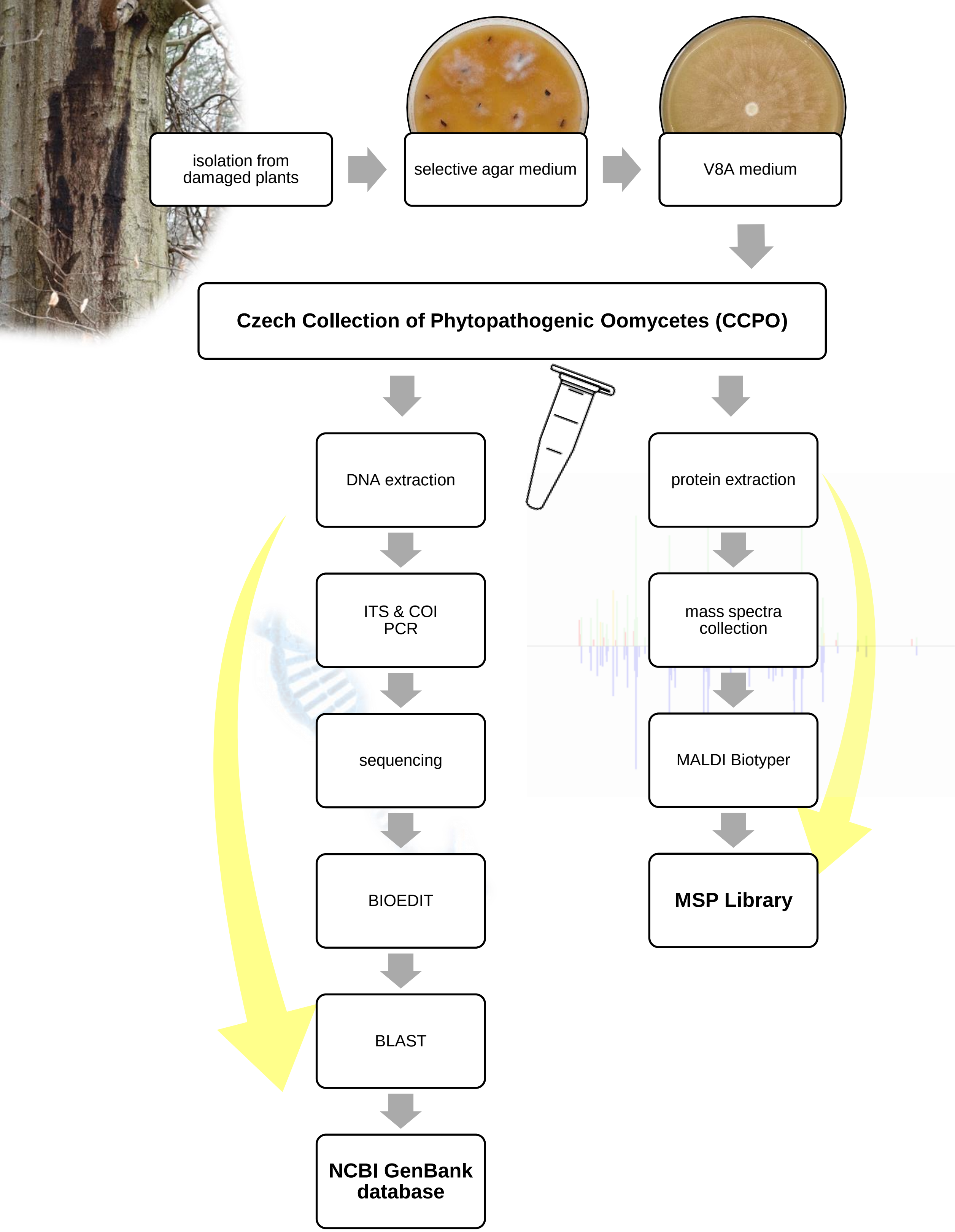
isolation from damaged plants
selective agar medium
V8A medium
Czech Collection of Phytopathogenic Oomycetes (CCPO)
DNA extraction
ITS & COI PCR
sequencing
BIOEDIT
BLAST
NCBI GenBank database
protein extraction
mass spectra collection
MALDI Biotyper
MSP Library
